# Supplementary material for: Encoding multistate charge order and chirality in endotaxial heterostructures
Source: Nat Commun. 2023 Sep 27;14:6031. doi: 10.1038/s41467-023-41780-y (PMC10533556; doi:10.1038/s41467-023-41780-y)
Supplement: Supplementary file 1 — Supplementary Information [file 41467_2023_41780_MOESM1_ESM.pdf]

Supplementary Information for

Encoding multistate charge order and chirality in endotaxial heterostructures

Samra Husremović[1], Berit H. Goodge[1,2], Matthew Erodici[1], Katherine Inzani[3], Alberto Mier[1], Stephanie M. Ribet[4,5,6], Karen C. Bustillo[4], Takashi Taniguchi[7], Kenji Watanabe[8], Colin Ophus[4], Sinéad M. Griffin[9,10], and D. Kwabena Bediako[1,11,*]

[1] *Department of Chemistry, University of California, Berkeley, CA 94720, USA*
[2] *Max-Planck-Institute for Chemical Physics of Solids, Nöthnitzer Str. 40, 01187, Dresden, Germany*
[3] *School of Chemistry, University of Nottingham, University Park, Nottingham NG7 2RD, United Kingdom*
[4] *National Center for Electron Microscopy, Molecular Foundry, Lawrence Berkeley National Laboratory, Berkeley, CA, USA*
[5] *Department of Materials Science and Engineering, Northwestern University, Evanston, Illinois 60208, United States*
[6] *International Institute of Nanotechnology, Northwestern University, Evanston, Illinois 60208, United States*
[7] *Research Center for Functional Materials, National Institute for Materials Science, Tsukuba 305-0044, Japan*
[8] *International Center for Materials Nanoarchitectonics, National Institute for Materials Science, Tsukuba 305-0044, Japan*
[9] *Materials Sciences Division, Lawrence Berkeley National Laboratory, Berkeley, California 94720, United States*
[10] *The Molecular Foundry, Lawrence Berkeley National Laboratory, Berkeley, California 94720, United States*
[11] *Chemical Sciences Division, Lawrence Berkeley National Laboratory, Berkeley, CA 94720, USA*
[*] *Correspondence to: bediako@berkeley.edu*

Supplementary Note 1: atomic force microscopy (AFM) of annealed TaS_2 flakes

Atomic force microscopy (AFM) images of annealed flakes (Figure 5c, Supplementary Figure 1) do not reveal a correlation between optical contrast steps and the flake height. This confirms that optical contrast (OC) changes do not result from thermally-induced changes in flake thickness.

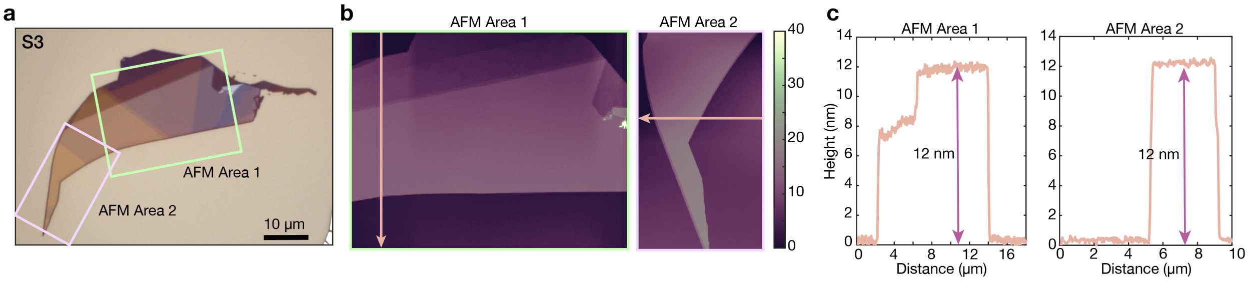

Supplementary Figure 1. Atomic force microscopy of annealed flakes. (a) Optical micrograph of flake S3 after thermal annealing. Areas where atomic force microscopy (AFM) maps were obtained are marked in colored rectangles. **(b)** AFM topography image of S3 in areas 1 and 2. **(c)** Height profiles along the orange lines from (b).

Supplementary Note 2: use of optical contrast measurements to quantify the number of H-$\mathrm{TaS_2}$ layers formed upon annealing $1T$–$\mathrm{TaS_2}$ crystals

Optical contrast of 2D flakes on SiO_2/Si is strongly influenced by their refractive index and absorption coefficient.[1,2] These intrinsic properties are shaped by the crystal structure and the resulting electronic structure.[3] Thus, the structurally distinct $1T$–TaS_2 and H-TaS_2 flakes on identical substrates exhibit significantly different optical contrasts.[4,5] We employ this property to quantify the number of H-TaS_2 layers formed upon annealing $1T$–TaS_2 crystals.

We found that the change in red optical contrast (ΔOCR) upon annealing is linearly related to the number of formed H-TaS_2 layers (Figure 1f). Here, ΔOCR is defined as:

$$\Delta OCR = CRD_{\text{after annealing}} - CRD_{\text{before annealing}} \tag{1}$$

$$CRD = (CR_{\text{flake}} - CR_{\text{substrate}})/CR_{\text{substrate}}) \tag{2}$$

where $CR_{\text{substrate}}$ and CR_{flake} are the red optical contrast values of the substrate and the flake, respectively.[5] Before annealing, CRD of $1T$–TaS_2 flakes is solely dependent their layer count (n). Thus, we can write the following expression:

$$CRD_{\text{before annealing}} = CRD_{1T\text{–TaS}_2}(n) \tag{3}$$

After annealing, x number of $1T$–TaS_2 layers is converted into H–TaS_2. Thus, we can write the red optical contrast after annealing as:

$$CRD_{\text{after annealing}} = CRD_{1T\text{–TaS}_2}(n-x) + CRD_{H\text{-TaS}_2}(x) \tag{4}$$

It follows from Equations 3 and 4 that ΔOCR can rewritten as:

$$\Delta OCR = CRD_{1T\text{–TaS}_2}(n-x) + CRD_{H\text{-TaS}_2}(x) - CRD_{1T\text{–TaS}_2}(n) \tag{5}$$

Note, $CRD_{1T\text{–TaS}_2}(n-x) \approx CRD_{1T\text{–TaS}_2}(n)$ if $x \lessapprox 6$ and $n \approx 12-30$ layers.[5] In this regime, we can rewrite Equation 5 as:

$$\Delta OCR \sim CRD_{H\text{-TaS}_2}(x) \tag{6}$$

Equation 6 elucidates why ΔOCR is linear with respect to the number of layers of H-TaS_2 layers (Figure 1f). Namely, $CRD_{H\text{-TaS}_2}(x)$ is linearly proportional to x if $x \lessapprox 6$,[4] making ΔOCR linearly proportional to x as well. Thus, based on Equations 5 and 6, the use of ΔOCR for estimating the number of H-TaS_2 layers in thermally-produced TaS_2 heterostructures is the most appropriate when annealing nano-thick $1T$–TaS_2 flakes that undergo a moderate degree of polytype transformation.

Supplementary Note 3: polymorph transformations in encapsulated samples

In this work, most flakes were annealed without hBN encapsulation to simplify ΔOCR analysis upon annealing. However, we found that thermally induced polytype transformation persists in fully encapsulated flakes, based on the sharpening of Raman modes after annealing (Supplementary Figure 2). Note that the preparation of hBN and $1T$–TaS_2 stacks was performed with the PC-PDMS stacking method used for the fabrication of c-axis TEM samples (See "Methods" section).

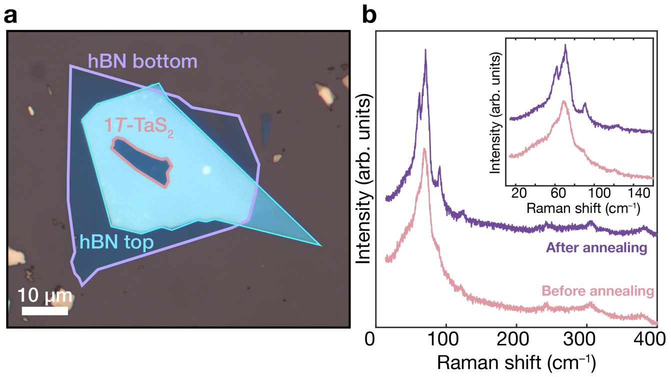

Supplementary Figure 2. Polytype transformations of fully encapsulated flakes. (a) Optical micrograph of a ∼12-layer $1T$–TaS_2 flake encapsulated with top and bottom hBN. Flake outlines are marked and false-colored. (**b**) Representative linearly polarized Raman spectrum of the hBN/TaS_2/hBN heterostructure before and after annealing. The inset displays a zoomed-in view of the ultra-low frequency (ULF) Raman modes.

Supplementary Note 4: cryogenic selected area electron diffraction (SAED)

We also obtained cryogenic SAED with the Gatan cryo non-transfer 636 holder for FEI. SAED, obtained upon warming from 100 K, does not evince significant changes in the 100 K–300 K range, affirming the absence of CDW phase transitions at cryogenic temperatures (Supplementary Figure 3).

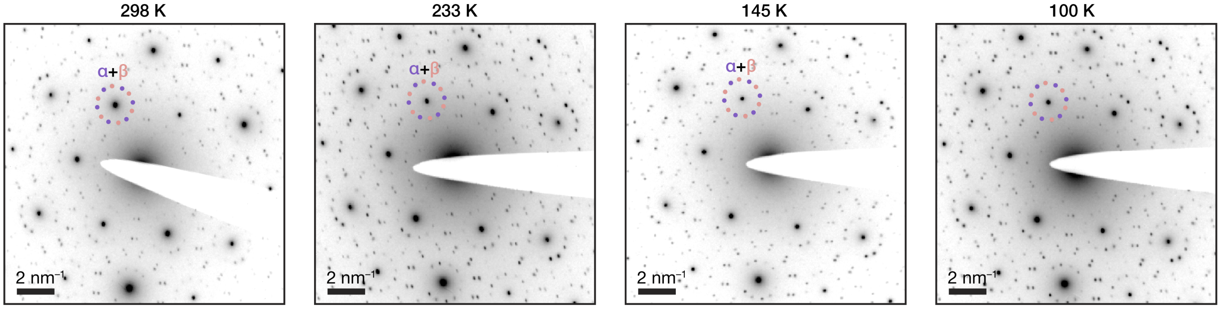

Supplementary Figure 3. Cryogenic selected area electron diffraction (SAED) of polytype heterostructures. SAED of a representative H–TaS_2/1T–TaS_2 heterostructure obtained upon warming from 100 K to 298 K.

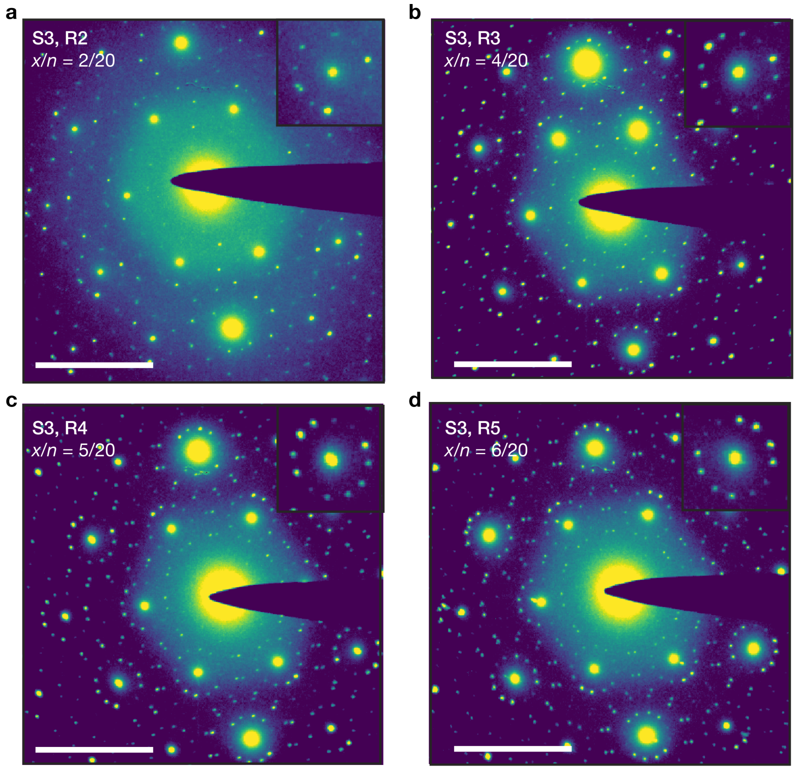

Supplementary Figure 4. Selected area electron diffraction (SAED) of heterostructures with different polytype compositions. (a–d) SAED patterns of flake S3 in regions R2 **(a)**, R3 **(b)**, R4 **(c)** and R5 **(d)**. Scale bars are 5 nm^{-1}.

Supplementary Note 5: high-resolution TEM (HRTEM)

High-resolution TEM was performed using the FEI ThemIS 60-300 STEM/TEM operated at 80 kV to investigate the CDW structure at the atomic scale. Fourier transform analysis of HRTEM micrographs (Supplementary Figure 5) revealed coeval α and β superstructure fringes across the entirety of the studied samples, further supporting that these heterochiral superstructures form in the out-of-plane direction.

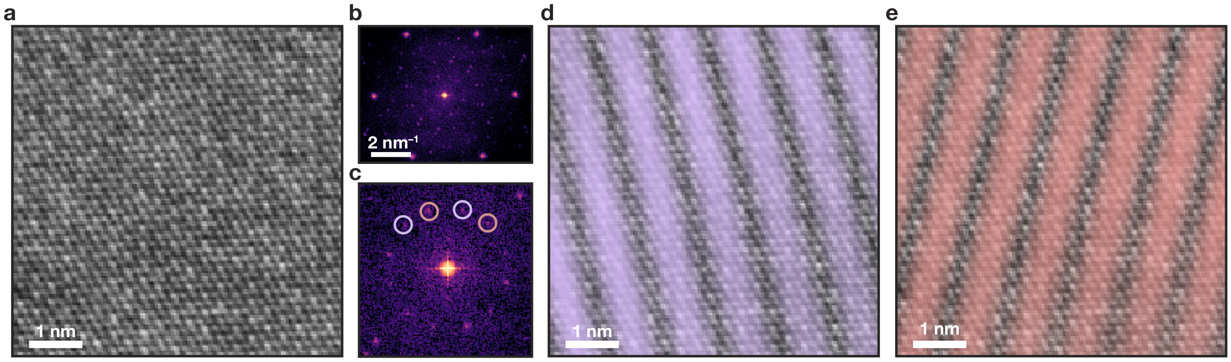

Supplementary Figure 5. High-resolution TEM of TaS_2 heterostructures. (a) HRTEM micrograph of a representative H–TaS_2/1T–TaS_2 heterostructure. (**b**) Fast Fourier transform (FFT) of (a). **(c)** Enlarged view of (b) in the vicinity of the zero frequency peak. **(d)** Overlay of (a) and the inverse FFT (iFFT) image generated from the components indicated by violet circles in (c). The iFFT (violet lines) represents the α fringes. **(e)** Overlay of (a) and the inverse FFT (iFFT) image from the components indicated by orange circles in (c). The iFFT (orange lines) represents the β fringes.

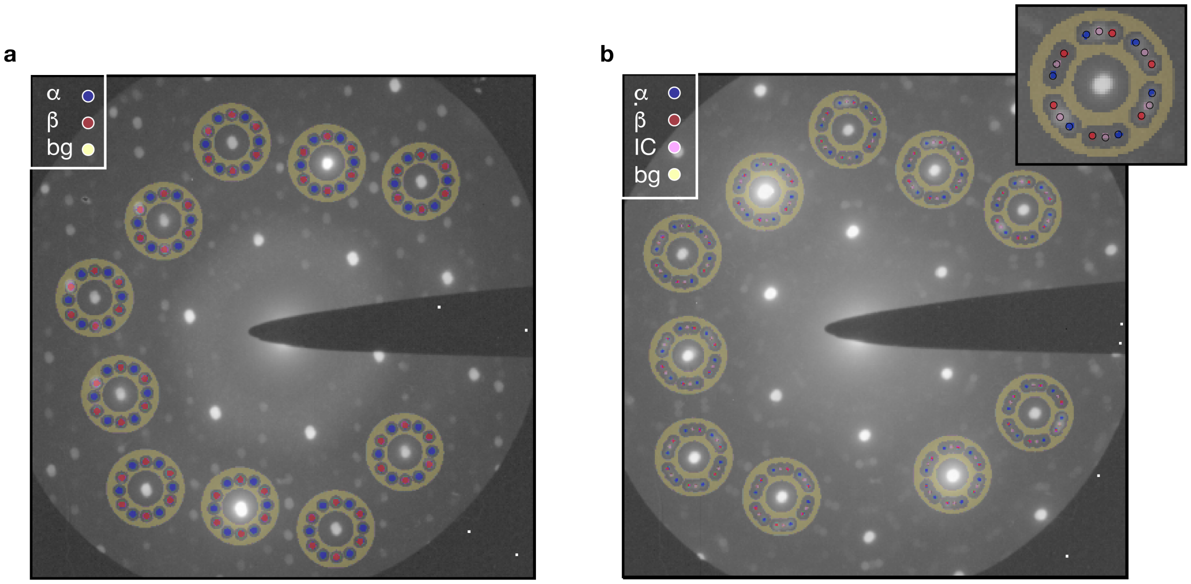

Supplementary Figure 6. Virtual detectors for analysis of four-dimensional scanning transmission electron microscopy (4D-STEM) data. (a) Maximal diffraction pattern, displayed on a logarithmic scale, overlaid with virtual detectors (masks) for: α (blue), β (red) and background–bg (yellow). Detectors from (a) were used for the analysis of room temperature 4D-STEM data. **(b)** Maximal diffraction pattern, displayed on a logarithmic scale, overlaid with virtual detectors for: α (blue), β (red), IC (pink), and background (yellow). These detectors were used for the analysis of temperature-dependent 4D-STEM data.

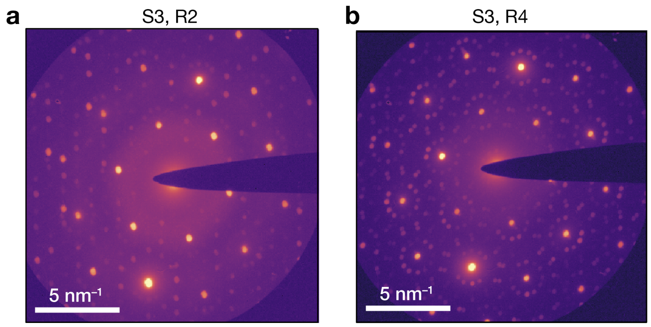

Supplementary Figure 7. Maximal diffraction patterns of sample S3. Maximal diffraction patterns, displayed on a logarithmic scale, of S3 in regions R2 **(a)** and R4 **(b)**. Scale bars are 5 nm^{-1}.

Supplementary Note 6: filtering of DPC-STEM micrographs

Raw DPC images (Supplementary Figure 8a), which are acquired simultaneously with high-angle annular dark-field (HAADF) STEM images (Supplementary Figure 8b), were filtered with a Gaussian high-pass (GHP) filter with $\sigma = 20$ pixels (Supplementary Figure 8c, Figure 1g–j). The resulting images were compared to micrographs filtered with $\sigma = 50$ pixels (Supplementary Figure 9) to confirm that our GHP filtering process does not introduce artifacts. The $\sigma = 20$ pixels images were used for assigning the TaS_2 polytype in our samples. The accuracy of these assignments was further verified by analyzing images that were thresholded using the Otsu filter (Supplementary Figure 10). We utilized the Python package SciPy[6] for bandpass filtering, while the thresholding analysis of images was conducted using the scikit-image[7] Python package.

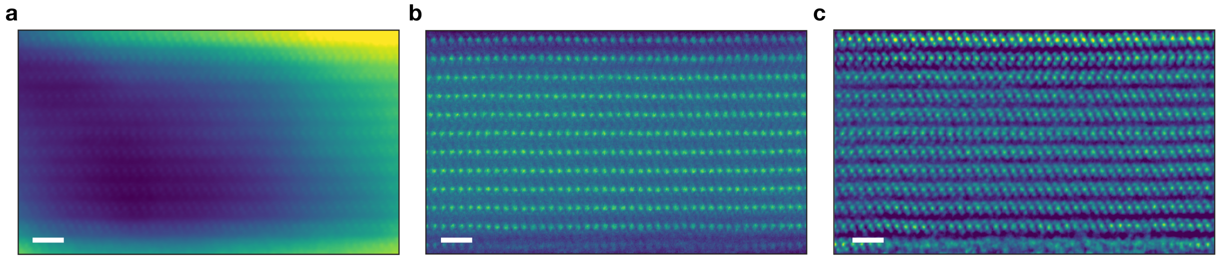

Supplementary Figure 8. Filtering of DPC-STEM data. (a) Raw DPC-STEM data. **(b)** Raw HAADF image. **(c)** DPC-STEM data after $\sigma = 20$ Gaussian high-pass filtering. Scale bars are 1 nm.

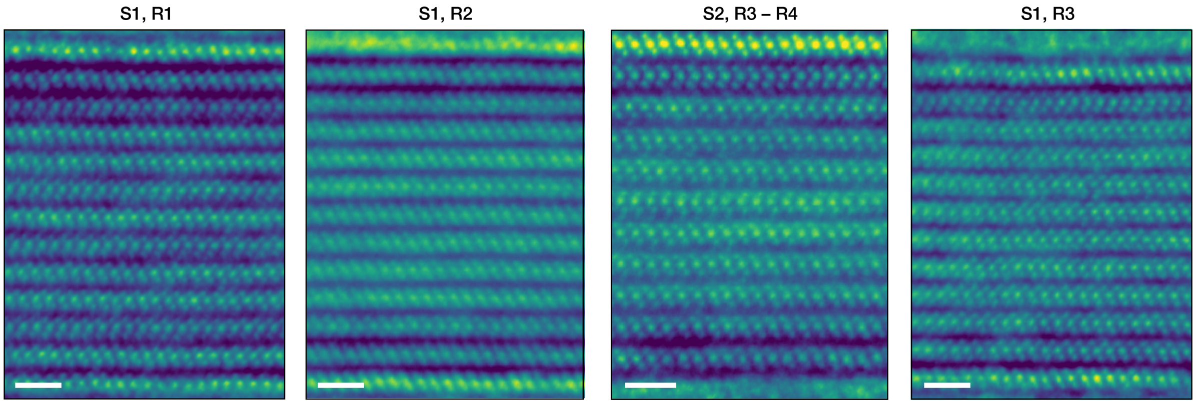

Supplementary Figure 9. DPC-STEM data of samples S1 and S2 filtered with a bandpass filter. DPC-STEM data after $\sigma = 50$ pixels Gaussian high-pass filtering. Scale bars are 1 nm.

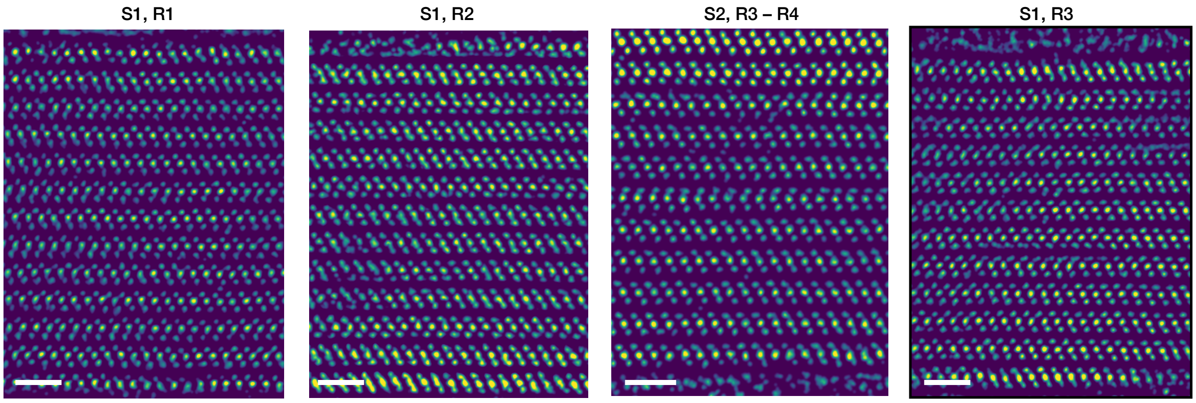

Supplementary Figure 10. DPC-STEM data of samples S1 and S2 filtered with a bandpass filter and thresholding. DPC-STEM data after Gaussian high-pass filtering and thresholding with Otsu's method. Scale bars are 1 nm.

Supplementary Note 7: determining polymorph from DPC-STEM micrographs

Polymorph composition was determined from DPC-STEM micrographs by analyzing the vertical chalcogen stacking within individual TaS_2 layers; S ions are parallel in H-TaS_2 and diagonal in the T-TaS_2 polytype (Supplementary Figure 11).

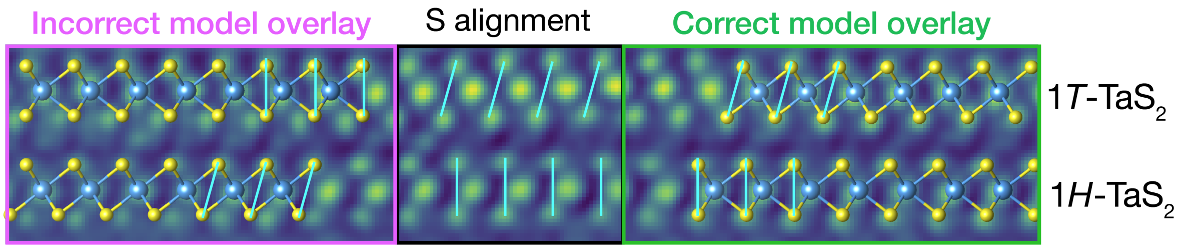

Supplementary Figure 11. Determining polymorphs from DPC-STEM micrographs. Filtered DPC-STEM micrograph along the $[10\bar{1}0]$ zone axis overlaid with crystallographic models for $1T$–TaS_2 (ref [8]) and H–TaS_2 (ref [9]). Neighboring S ions in the data were connected with blue lines for the ease of discriminating between the H–TaS_2 and $1T$–TaS_2 polymorphs, whose S ions are parallel and diagonal, respectively. If an incorrect polytype is overlaid with the data (left), sulfur ions of the model and the data do not overlap. The opposite holds if the correct polytype is superimposed with the data (right).

Supplementary Note 8: Raman spectroscopy setup

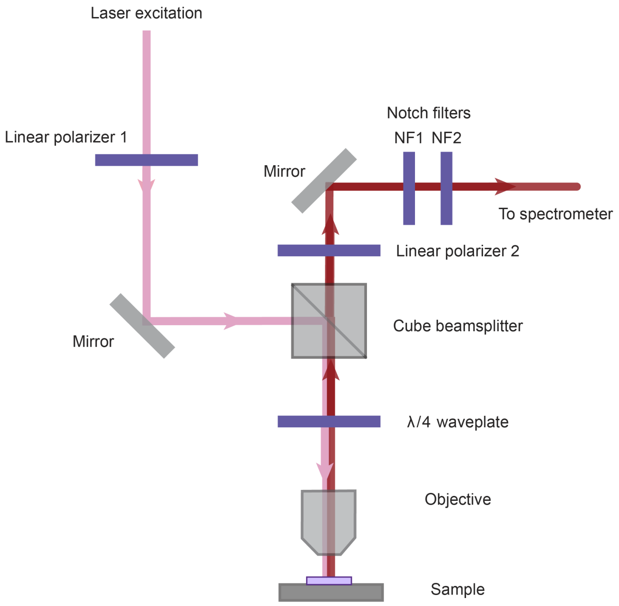

Supplementary Figure 12. Setup for circularly polarized Raman measurements. Illustration of the Raman setup for circularly polarized measurements using two linear polarizers.

Herein, the procedure for setting up circularly polarized Raman measurements is detailed. First, linear polarizer 1 (LP1) was inserted into the optical path, and its transmission axis was found by looking for the angle corresponding to the maximum Raman intensity of the 520.7 cm^{-1} peak for a silicon reference sample. LP1 was kept at this angle of maximum transmission for all subsequent measurements. Second, linear polarizer 2 (LP2) was inserted, and its transmission axis was found by rotating the polarizer until achieving the maximum Raman intensity of the Si peak. In this configuration, LP1 and LP2 are in 'parallel'. Correspondingly, the angle of minimum transmission was found orthogonal to the transmission axis (i.e. LP1 and LP2 in the 'cross' configuration). Third, the $\lambda/4$ waveplate was inserted, and its fast and slow axes were determined by examining the transmission of the laser through a Wollaston prism. Note, the prism was inserted in place of the silicon reference, and an index card was placed below it. The fast and slow axes corresponded to angles on the $\lambda/4$ waveplate at which there was only one laser spot on the card (i.e. corresponding to either fully P- or S-polarization of the light, as the laser remains linearly polarized when traveling through either the fast or slow axis of the $\lambda/4$ waveplate). After this, the $\lambda/4$ waveplate was

aligned to the fast/slow axis and then rotated ±45° from this angle to generate circularly polarized light (σ^+= +45° and σ^-= -45°). Lastly, the circular polarization was verified by observing two equal-intensity spots of P- and S-polarization through the Wollaston prism regardless of the rotation angle of the prism.

To ensure a contrarotating configuration ($\sigma^+\sigma^-$ and $\sigma^-\sigma^+$), where $\sigma^i\sigma^s$ refers to the circular polarization of the incident (σ^i) and scattered (σ^s) light, the orientation of LP2 was kept in 'cross' relative to LP1. On the other hand, the orientation of LP2 is kept in 'parallel' relative to LP1 when sampling corotating configurations (i.e. $\sigma^+\sigma^+$ and $\sigma^-\sigma^-$). Importantly, the orientation of LP2 only filters the type of Raman scattered light being sampled in the given measurement and *does not* preclude the excitation of the Raman-active modes themselves. The excitation of Raman modes only depends on the type of circular polarization of the incident beam (σ^i).

Supplementary Note 9: Raman optical activity of TaS_2 heterostructures

Raman optical activity (ROA) can be understood by considering the interaction of circularly polarized light with Raman tensors, assigned based on the material's symmetry. In the C-CDW state, the space group for native $1T$–TaS_2 is $P\overline{3}$ (point group $C_{3\mathrm{i}}$).[10,11] However, H–TaS_2/$1T$–TaS_2 heterostructures are more accurately described by the $P3$ space group (C_3 point group), because H–TaS_2 layers break inversion symmetry. Nevertheless, the $C_{3\mathrm{i}}$ and C_3 point groups have identical Raman tensors:[12–14]

$$\mathbf{R}_{A_\mathrm{g}} = \begin{bmatrix} a & 0 & 0 \\ 0 & a & 0 \\ 0 & 0 & b \end{bmatrix} \quad \mathbf{R}_{{}^1E_\mathrm{g}} = \begin{bmatrix} c & d & e \\ d & -c & f \\ e & f & \cdot \end{bmatrix} \quad \mathbf{R}_{{}^2E_\mathrm{g}} = \begin{bmatrix} d & -c & -f \\ -c & -d & e \\ -f & e & \cdot \end{bmatrix}$$

Note, since our experiments are conducted in the back-scattering geometry with normal incidence, the relevant Raman tensor elements are:

$$\mathbf{R}_{A_\mathrm{g}} = \begin{bmatrix} a & 0 \\ 0 & a \end{bmatrix} \quad \mathbf{R}_{{}^1E_\mathrm{g}} = \begin{bmatrix} c & d \\ d & -c \end{bmatrix} \quad \mathbf{R}_{{}^2E_\mathrm{g}} = \begin{bmatrix} d & -c \\ -c & -d \end{bmatrix}$$

The presence of both diagonal and off-diagonal Raman tensor elements predisposes E_g modes to exhibit different intensities in the $\sigma^+\sigma^-$ and $\sigma^-\sigma^+$ measurement geometries (*i.e.* ROA), where $\sigma^i\sigma^s$ $(i, s = +/-)$ are phonon helicities of the incident and scattered light. Thus, interaction of E_g modes with circularly polarized light gives rise to the ROA of TaS_2 heterostructures. This is mathematically demonstrated below.

Raman intensity of a phonon mode with the Raman tensor $\mathbf{R}$ is proportional to $|\mathbf{e}_s^*\mathbf{R}\mathbf{e}_i|^2$, where $\mathbf{e}_s$ and $\mathbf{e}_i$ are the polarization vectors for the incident and scattered light, respectively. The polarization (Jones) vectors for the circularly polarized light can be described as:

$$\mathbf{e}_{\sigma^+} = \frac{1}{\sqrt{2}} \begin{bmatrix} 1 \\ i \end{bmatrix} \quad \mathbf{e}_{\sigma^-} = \frac{1}{\sqrt{2}} \begin{bmatrix} 1 \\ -i \end{bmatrix}$$

Thus, the intensity of the ${}^1E_\mathrm{g}$ modes in the two measuring geometries can be written as:

$$I_{\sigma^+\sigma^-} = c^2 - d^2 + 2cdi \quad \text{and} \quad I_{\sigma^-\sigma^+} = c^2 - d^2 - 2cdi$$

While the intensity of the ${}^2E_\mathrm{g}$ modes in the two measuring geometries can be written as:

$$I_{\sigma^+\sigma^-} = d^2 - c^2 - 2cdi \quad \text{and} \quad I_{\sigma^-\sigma^+} = d^2 - c^2 + 2cdi$$

As seen in the equations above, the intensity of E_g modes differs in the two $\sigma^i\sigma^s$ geometries, and E_g modes exhibit ROA. Further, the intensity of ${}^1E_\mathrm{g}$ and ${}^2E_\mathrm{g}$ modes changes in an opposite fashion; if ${}^1E_\mathrm{g}$ modes are more intense in $\sigma^+\sigma^-$, then ${}^2E_\mathrm{g}$ modes are less intense in $\sigma^+\sigma^-$. This inverse relationship stems from the opposite sign of the off-diagonal tensor elements for ${}^1E_\mathrm{g}$ and ${}^2E_\mathrm{g}$ modes. We note that ROA cannot be achieved in the corotating configurations ($\sigma^+\sigma^+/\sigma^-\sigma^-$) in which Raman modes display identical intensities (Supplementary Figure 13a)

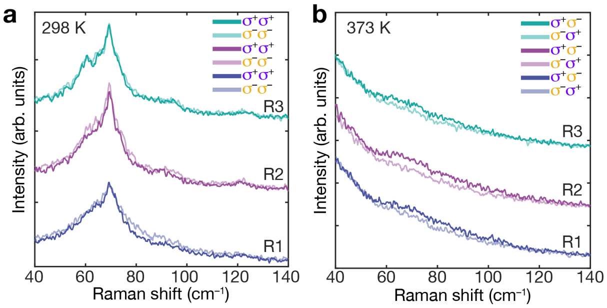

Supplementary Figure 13. Raman spectra of sample S4. **(a)** Raman spectra in the circular corotating polarization configurations ($\sigma^+\sigma^+$ and $\sigma^-\sigma^-$) obtained in regions R1–R3 of sample S4 at room temperature. **(b)** Raman spectra in the circular contrarotating polarization configurations ($\sigma^+\sigma^-$ and $\sigma^-\sigma^+$) obtained in regions R1–R3 of sample S4 at 373 K.

The ROA of TaS_2 heterostructures vanishes at high temperatures when the system enters

the IC-CDW phase (Supplementary Figure 13b). In the IC-CDW state, the space group for $1T$–TaS_2 is $P\overline{3}m1$ (point group D_{3d}). However, the overall heterostructure is more accurately described by the $P3m1$ space group (C_{3v} point group) due to inversion symmetry breaking by the H–TaS_2 layers. The Raman tensors for D_{3d} and C_{3v} do not contain both diagonal and off-diagonal components. Thus, ROA cannot be achieved.[12–14]

We note that ROA can also be used to assign the overall chirality of TaS_2 heterostructures.[11] Since α and β have opposite mirror symmetry, their Raman tensors also exhibit opposite mirror symmetry. Note, a mirror symmetry operation on a Raman tensor $\left(\begin{smallmatrix} a & c \\ c & b \end{smallmatrix}\right)$ yields $\left(\begin{smallmatrix} a & -c \\ -c & b \end{smallmatrix}\right)$.[11] As follows, ROA is opposite for the two superstructures; if a chosen $\sigma^i\sigma^s$ leads to intense ^1E_g modes for α, then the same measurement geometry will lead to less intense ^1E_g modes for β. In reference[11], a combination of Raman measurements and scanning tunneling microscopy (STM) was used to correlate the Raman response to the superlattice structure in bulk $1T$–TaS_2, which displays exclusively α or β. The correlation from reference[11] can be used to assign the overall chirality in our heterostructures, which contain a mixture of α and β. Supplementary Table 1 summarizes the process of identifying the overall chirality of TaS_2 heterostructures.

Supplementary Table 1: Process of identifying the chirality of TaS_2 heterostructures based reference[11].

Measurement	$E_g(I)$	$E_g(II)$	Overall chirality
$\sigma^+\sigma^-$	Less intense	More intense	α (L)
$\sigma^-\sigma^+$	More intense	Less intense	α (L)
$\sigma^+\sigma^-$	More intense	Less intense	β (R)
$\sigma^-\sigma^+$	Less intense	More intense	β (R)

Note, $E_g(I)$ and $E_g(II)$ modes display opposite ROA and were grouped by symmetry (Figure 3 and Supplementary Figure 14).

Supplementary Note 10: filtering of Raman data

Linearly polarized Raman data in the main paper was smoothed with a Savitzky-Golay filter. Circularly polarized data was not filtered.

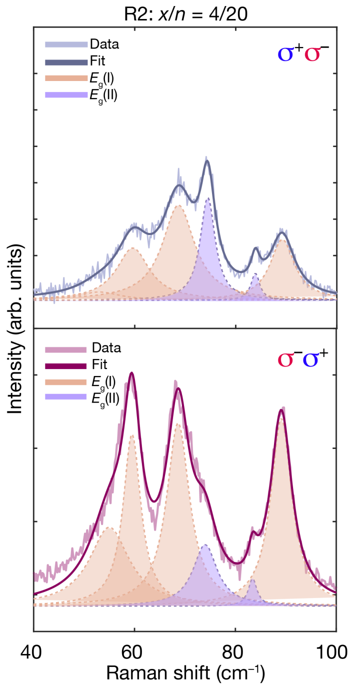

Supplementary Figure 14. Circularly polarized Raman response of sample S4 in region R2. Raman spectra in the circular contrarotating polarization configurations ($\sigma^+\sigma^-$ and $\sigma^-\sigma^+$) obtained in region R2 of a 20-layer sample S4. Lorentzian peak fits and the cumulative fits are displayed.

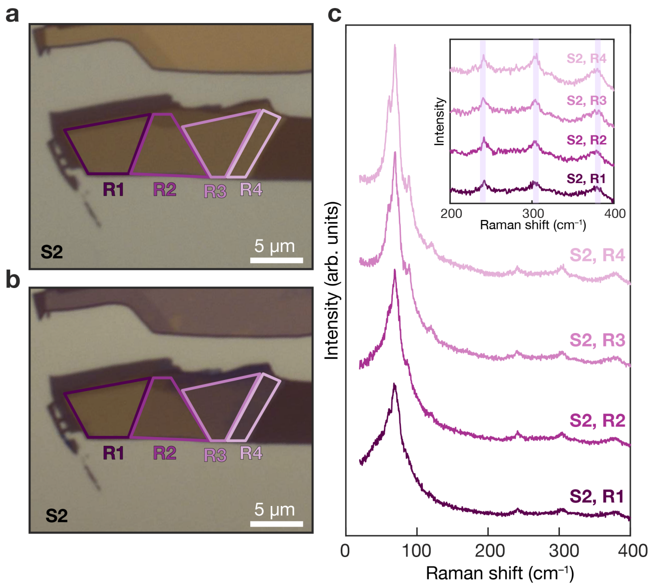

Supplementary Figure 15. Thermally induced polytype and CDW transformations of sample S2. Optical micrographs of flake S2 before **(a)** and after **(b)** thermal annealing. **(c)** Raman spectra in different regions of S2 after annealing.

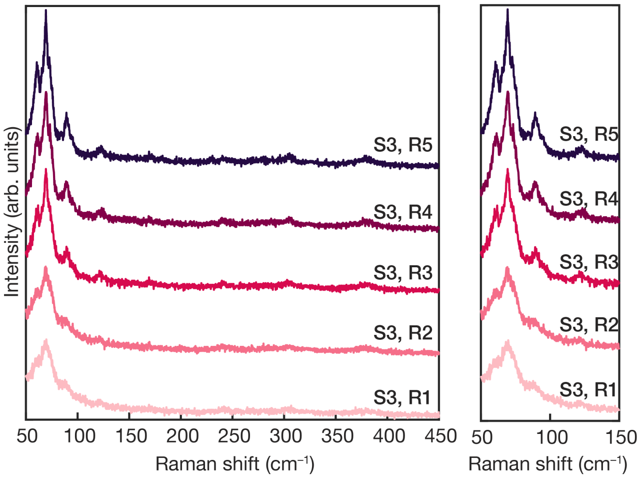

Supplementary Figure 16. Raman response of sample S3. Linearly polarized Raman spectra of flake S3 taken in regions R1–R5 after thermal annealing.

Supplementary Note 11: reproducibility of multistep CDW transitions

Multiple thermal sweeps were performed for mesoscopic devices fabricated from S1 (Supplementary Figure 17) and S4 (Supplementary Figure 18, Figure 4d) to examine the reproducibility of the ladder resistance steps. These measurements revealed that the stepwise resistance changes are highly reproducible upon repeated thermal cycling.

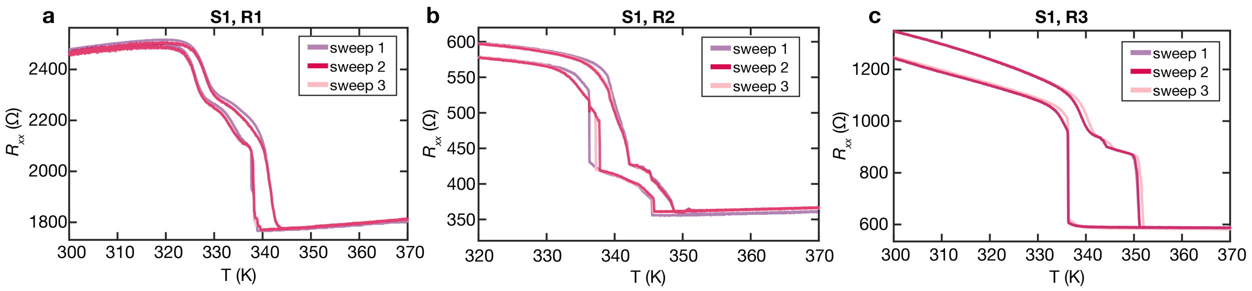

Supplementary Figure 17. Reproducibility of resistance profiles for sample S1. (a–c) Resistance of sample S1 in regions R1 **(a)**, R2 **(b)** and R3 **(c)** across three temperature sweeps. The sweep rate was kept constant at 1 K/min for all thermal cycles.

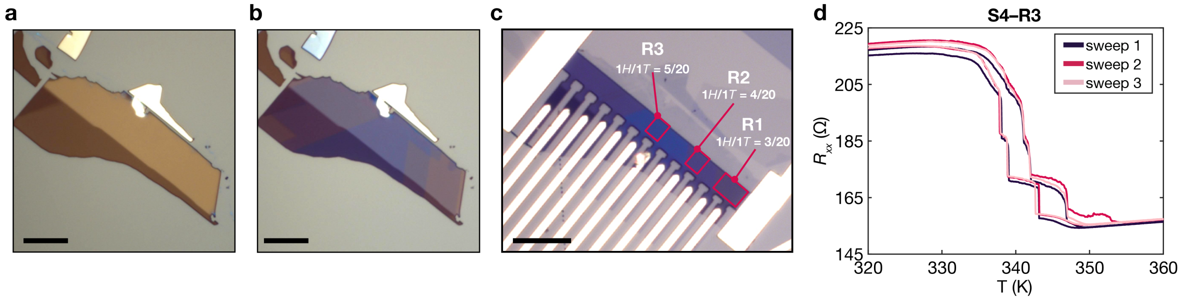

Supplementary Figure 18. Polytype transformations and transport measurements of sample S4. (a–c) Optical micrograph of S4 before annealing **(a)**, after annealing **(b)**, and after nanofabrication **(c)**. Regions of interest are marked. **(d)** Temperature-dependent resistance of S4 in region R3 across three thermal sweeps. The temperature sweep rate was 1 K/min.

Supplementary Note 12: CDW order parameters simulations

CDW state of $1T$–TaS_2 is characterized by the real-space order parameter described by Equation 7:

$$\delta\rho(\mathbf{r}) = \rho_0 \sum_{j=1}^{j=3} cos(\mathbf{q_j} \cdot \mathbf{r} + \phi_j(\mathbf{r})) \tag{7}$$

Here, $\mathbf{q_j}$ is the CDW wave vector, ρ_0 is the amplitude of the CDW order parameter and $\phi_j(\mathbf{r})$ the CDW phase, describing the location of the charge modulation in reference to the underlying host lattice.[15,16] For simplification, we calculated the CDW order parameter with $\phi_j(\mathbf{r}) = 0$, which is appropriate for the CCDW phase.[17–19] In Figure 19, we examine the charge distribution in α–α (Figure 19a) and α–β (Figure 19b) vertically stacked CDW superlattices by combining their respective real-space CDW order parameters (Supplementary Figure 19 c–f). Note that the interlayer slip across the H–TaS_2 interface was accounted for. The charge distribution for different CDW vertical arrangements is distinct (Supplementary Figure 19c–j), which is in agreement with the literature.[20]

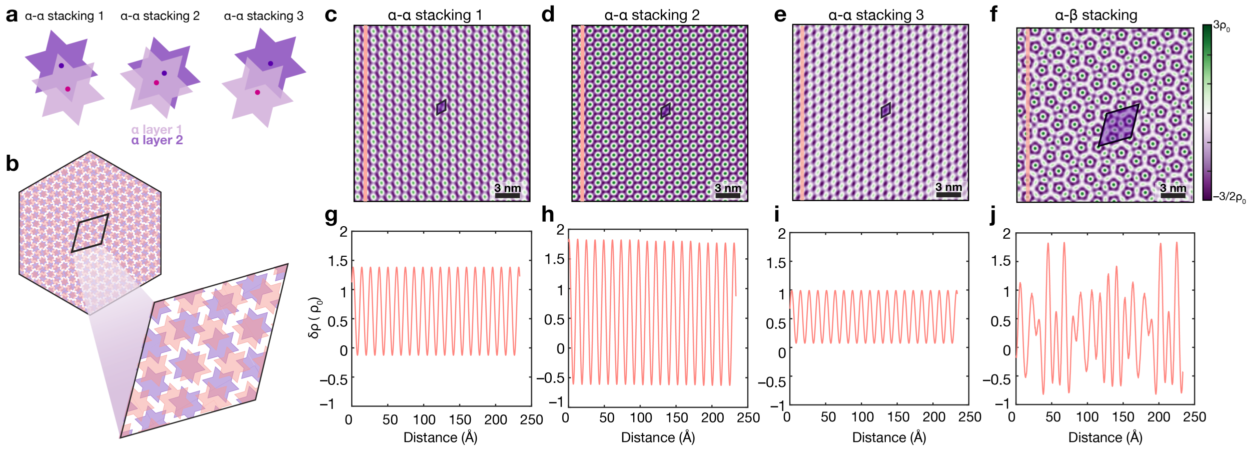

Supplementary Figure 19. Modeling of the CDW order parameter in TaS_2 heterostructures. (a) Non-degenerate SOD stacking arrangements for the α–α out-of-plane superstructures. **(b)** Stacking arrangements for α–β out-of-plane superlattices. Unit cell of the CDW moiré superstructure is indicated in black. Real-space CDW order parameter ($\delta\rho(\mathbf{r})$) simulated for the following out-of-plane arrangements: **(c)** α–α stacking 1, **(d)** α–α stacking 2, **(e)** α–α stacking 3 and **(f)** α–β stacking. Violet-shaded regions represent the unit cell of the CDW moiré superstructure. **(g–j)** Line profiles of (c)–(f) in regions marked with orange lines.

Supplementary Note 13: further insights from thermal annealing studies

In the thermal annealing studies, we find that the largest ΔOCR tends to localize around macroscopic flake defects (Supplementary Figure 20). Further, upon multiple annealing cycles, TaS_2 heterostructures become increasingly laterally homogeneous (Supplementary Figure 21). Lastly, we find the polytype transformation onsets at 230 °C, which is consistent with literature precedent.[21]

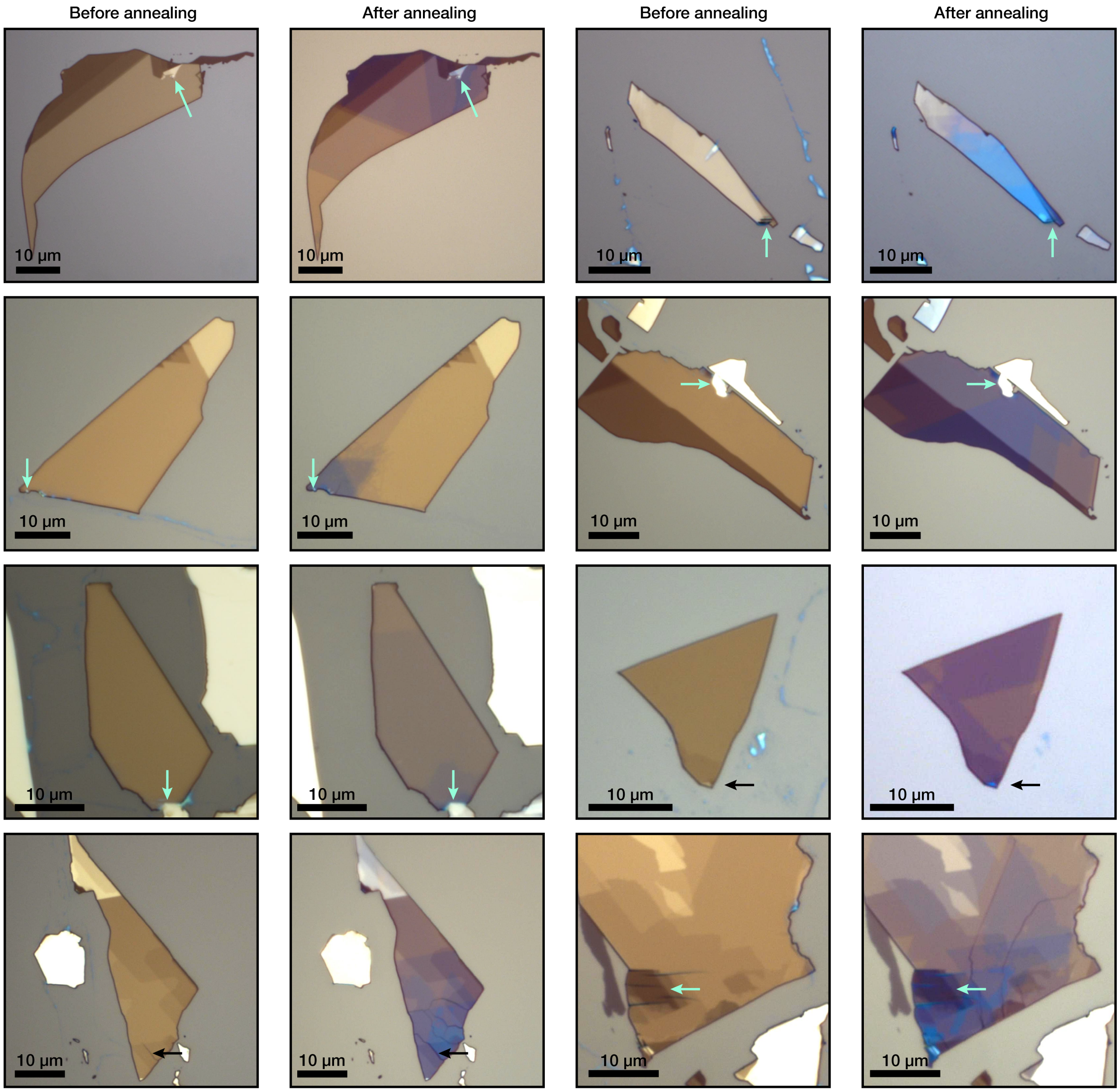

Supplementary Figure 20. Nucleation of polytype transformations at macroscopic flake defects. Optical micrographs of flakes before and after thermal annealing at 350 °C for 30 minutes. Arrows point to macroscopic flake defects (folds, wrinkles).

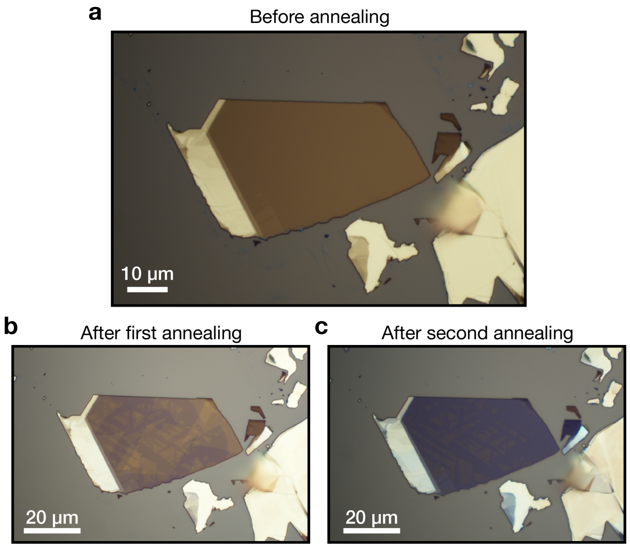

Supplementary Figure 21. Polytype transformations with successive heating cycles. (a–c) Optical micrographs of a $1T$–TaS_2 flake before annealing **(a)**, after the first **(b)** and after the second **(c)** 30 minute annealing cycle at 350 °C.

Supplementary References

1. Jung, I. et al. Simple Approach for High-Contrast Optical Imaging and Characterization of Graphene-Based Sheets. *Nano Letters* **7,** 3569–3575. ISSN: 1530-6984 (2007).
2. Munkhbat, B., Wróbel, P., Antosiewicz, T. J. & Shegai, T. O. Optical Constants of Several Multilayer Transition Metal Dichalcogenides Measured by Spectroscopic Ellipsometry in the 300–1700 nm Range: High Index, Anisotropy, and Hyperbolicity. *ACS Photonics* **9,** 2398–2407 (2022).
3. Newnham, R. E. in *Properties of Materials: Anisotropy, Symmetry, Structure* (Oxford University Press, 2004). ISBN: 9780198520757.
4. Husremović, S. et al. Hard ferromagnetism down to the thinnest limit of iron-intercalated tantalum disulfide. *Journal of the American Chemical Society* **144,** 12167–12176 (2022).
5. Li, H. et al. Rapid and reliable thickness identification of two-dimensional nanosheets using optical microscopy. *ACS Nano* **7,** 10344–10353 (2013).
6. Virtanen, P. et al. SciPy 1.0: Fundamental Algorithms for Scientific Computing in Python. *Nature Methods* **17,** 261–272 (2020).
7. Van der Walt, S. et al. scikit-image: image processing in Python. *PeerJ* **2,** e453 (2014).
8. Spijkerman, A., de Boer, J. L., Meetsma, A., Wiegers, G. A. & van Smaalen, S. X-ray crystal-structure refinement of the nearly commensurate phase of $1T$-TaS_2 in (3+2)-dimensional superspace. *Physical Review B* **56,** 13757 (1997).
9. Johnston, D. & Keelan, B. Superconductivity and magnetism of $M_x(H_2O)_yTaS_2$ layered cointercalation compounds. *Solid State Communications* **52,** 631–634 (1984).
10. Von Witte, G. et al. Surface structure and stacking of the commensurate $(\sqrt{13} \times \sqrt{13})R13.9^\circ$ charge density wave phase of $1T - TaS_2(0001)$. *Physical Review B* **100,** 155407 (2019).
11. Yang, H. F. et al. Visualization of Chiral Electronic Structure and Anomalous Optical Response in a Material with Chiral Charge Density Waves. *Physical Review Letters* **129,** 156401 (2022).

12. Aroyo, M. I. et al. Bilbao Crystallographic Server: I. Databases and crystallographic computing programs. *Zeitschrift für Kristallographie-Crystalline Materials* **221,** 15–27 (2006).

13. Aroyo, M. I., Kirov, A., Capillas, C., Perez-Mato, J. & Wondratschek, H. Bilbao Crystallographic Server. II. Representations of crystallographic point groups and space groups. *Acta Crystallographica Section A: Foundations of Crystallography* **62,** 115–128 (2006).

14. Aroyo, M. I. et al. Crystallography online: Bilbao crystallographic server. *Bulg. Chem. Commun* **43,** 183–197 (2011).

15. Chatterjee, U. et al. Emergence of coherence in the charge-density wave state of 2H-$NbSe_2$. *Nature Communications* **6,** 1–7 (2015).

16. Jarnac, A. et al. Photoinduced charge density wave phase in 1T-TaS_2: growth and coarsening mechanisms. *Comptes Rendus. Physique* **22,** 139–160 (2021).

17. Chen, C. *On the Nature of Charge Density Waves, Superconductivity and Their Interplay in 1T-$TiSe_2$* (Springer Nature, 2019).

18. Tsen, A. W. et al. Structure and control of charge density waves in two-dimensional 1T-TaS_2. *Proceedings of the National Academy of Sciences* **112,** 15054–15059 (2015).

19. Dai, J. et al. Microscopic evidence for strong periodic lattice distortion in two-dimensional charge-density wave systems. *Physical Review B* **89,** 165140 (2014).

20. Liu, L. et al. Unveiling Electronic Behaviors in Heterochiral Charge-Density-Wave Twisted Stacking Materials with 1.25 nm Unit Dependence. *ACS Nano.* ISSN: 1936-0851 (2023).

21. Wang, Z. et al. Surface-limited superconducting phase transition on 1T-TaS_2. *ACS Nano* **12,** 12619–12628 (2018).
